# Virtual Coaching Delivered by Pharmacists to Prevent COVID-19 Transmission

**DOI:** 10.1177/00185787211032354

**Published:** 2021-07-10

**Authors:** Derar H. Abdel-Qader, Ahmad Z. Al Meslamani, Nadia Al Mazrouei, Asma A. El-Shara, Husam El Sharu, Eman Merghani Ali, Samah Bahy Mohammed Ebaed, Osama Mohamed Ibrahim

**Affiliations:** 1 University of Petra, Amman, Jordan; 2 Al Ain University of Science and Technology, Abu Dhabi, United Arab Emirates; 3 University of Sharjah, Sharjah, United Arab Emirates; 4 Philadelphia University, Amman, Jordan; 5 Indiana University Center for Health Innovation and Implementation Science, Indianapolis, Indiana, USA; 6 Jazan University, Jazan, Saudi Arabia; 7 Benha University, Benha, Egypt; 8 Cairo University, Giza, Egypt

**Keywords:** pharmacists, education, medication safety, residency training, programs

## Abstract

**Background:** While the role of pharmacists in the current pandemic control has been recognized worldwide, their coaching efforts to improve public’s behaviors that could prevent COVID-19 transmission has been rarely investigated. **Objectives:** To assess whether pharmacist-based virtual health coaching sessions could increase the proportion of people who practised healthy social behaviors, to test whether this model can increase the public acceptance of COVID-19 vaccines, and to measure whether these behaviors could actually prevent contracting COVID-19. **Method:** In this randomized controlled trial, adults who matched specific criteria were randomly allocated into 2 arms. The active arm received 12 pharmacist-based virtual coaching sessions delivered via Zoom^®^ over a month. Participants allocated to the control arm received no coaching. At the end of the last coaching session, both groups were asked to complete a structured questionnaire for outcome assessment. Participants in the active group were followed up to 2 weeks after the end of the last coaching session to check if they contracted COVID-19 or not. The SPSS software version 26.0 (IBM Corp., Chicago, IL) was used for statistical analysis. **Results:** Of the 300 participants who gave consent for participation, 295 completed the study (147 from the active arm and 148 from the control arm). The proportion of those using face masks, avoiding crowds, and willing to be isolated if infected in the active arm was increased from 51.70%, 53.74%, and 59.86% at baseline to 91.83%, 80.27%, and 96.59% at the end of coaching, respectively (all with *P* < .05). In addition, the proportion of behaviors, such as disinfecting surfaces, not touching the T-zone, and avoid sharing personal belongings with colleagues at work was increased from 36.05%, 27.89%, and 46.93% at baseline to 63.94%, 52.38%, and 87.75% at the end of coaching, respectively (all with *P* < .05). Avoid touching the T-zone (OR = 0.43; 95% CI, 0.24-0.89) and using disposable tissues (OR = 0.30; 95% CI, 0.18-0.77), each versus using face masks appropriately were more likely to get COVID-19. **Conclusion:** Pharmacist-based virtual health coaching could be a potential strategy to increase the proportion of behaviors that could curtail the spread of COVID-19.

## Introduction

More than a year ago, the World health Organization (WHO) declared the Coronavirus Disease 2019 (COVID-19) a public health emergency of international concern.^1,2^ The new virus can be transmitted through respiratory aerosols of infected individuals. Additionally, it can be transmitted by touching contaminated surfaces, and proceed to touch the T-zone (nose, mouth, and eyes).^3,4^ Epidemiological studies in the Middle East reported an accelerated spread of the virus.^2,3^ The case was the same in Jordan, as an Eastern Mediterranean country, that shares borders with Syria, Iraq, Saudi Arabia, and Palestine (CIA.gov; Hamed, 2020).

In the light of the emerging necessities to enhance pharmacist’s role in raising awareness and assist their patients, novel approaches have to be adopted.^5^ A new concept was found positively motivate patients in behaviors modification.^5^ Health coaching has been implemented by pharmacists, showing promising results in patient-centered education and counseling, patient compliance, accountability, and improving long term conditions, in addition to cost benefits.^5-7^ Some countries offer health coaching services, such as, the US, Canada, UK, and Netherland.^6^ In 2020, an Australian study has shown that community pharmacists are facing obstacles in implementing health coaching as they need more training.^6^

During COVID-19 pandemic, the International Federation of Pharmacists (FIP) released guidelines for pharmacists and pharmacy workforce to operate in a safely manner.^8^ As pharmacists are considered the most accessible health-care providers, they have a pivotal role in controlling the spread of coronavirus pandemic.^8-11^

In Jordan, the first case was reported on March 2, 2020, and within less than 2 weeks, to contain the spread of the virus, Jordanian authorities declared a state of emergency and activated a lockdown, including closing borders, educational, and religious institutes.^5-8^ The COVID-19 statistical report of Jordanian Ministry of Health until 6th of June 2021 confirmed 739 847 cumulative positive COVID-19 cases, as well as 9301 active cases, and 9530 total deaths.^9^ Two weeks after documenting the first case, the government in Jordan activated the Defense Law, which authorizes the Minister of Defence to issue orders based on the situation.^12^ Consequently, international borders were closed, movements between cities were suspended except for key workers, and public health measures to control the infection were enforced. The new SARS-CoV-2 B.1.1.7 variant (20I/501Y.V1, also called variant of concern VOC 202012/01) initially detected in the UK has rapidly expanded its geographical range to other countries,^13^ including Jordan. A small-scale genome sequencing initiative conducted in Jordan reported that around 70% of all SARS-CoV-2 detections were B.1.1.7 viruses. The presence of B.1.1.7 variant on the territory, however, poses critical challenges to epidemic control. Its higher transmissibility represents a strong selective advantage to have rapidly become the dominant strain in Jordan. Although pharmacists are trusted by the vast majority of the public, pharmacists’ role in Jordan focuses on dispensing medications and the patient-centered care provided is still limited given the absence of continuing development programs and lack of supportive regulations.^14,15^ Pharmacists in Jordan are engaged in four areas; hospital settings, community settings, industrial settings, and marketing area.^16,17^ The Jordanian pharmacists’ association (JPA) indicated that 25 700 pharmacists are licensed in Jordan, of which the vast majority work in community settings.^18^ Integration of health technology (ie, telepharmacy) to pharmacy practice is still in its beginnings.^19^

Accordingly, this study aimed to test if virtual coaching delivered by community pharmacists can be efficient in reducing the spread of the current pandemic among people in Jordan by enhancing their health behaviors and if this approach can influence the public perception about immunization.

## Methods

### Trial Design and Sample Size

**Figure 1.**
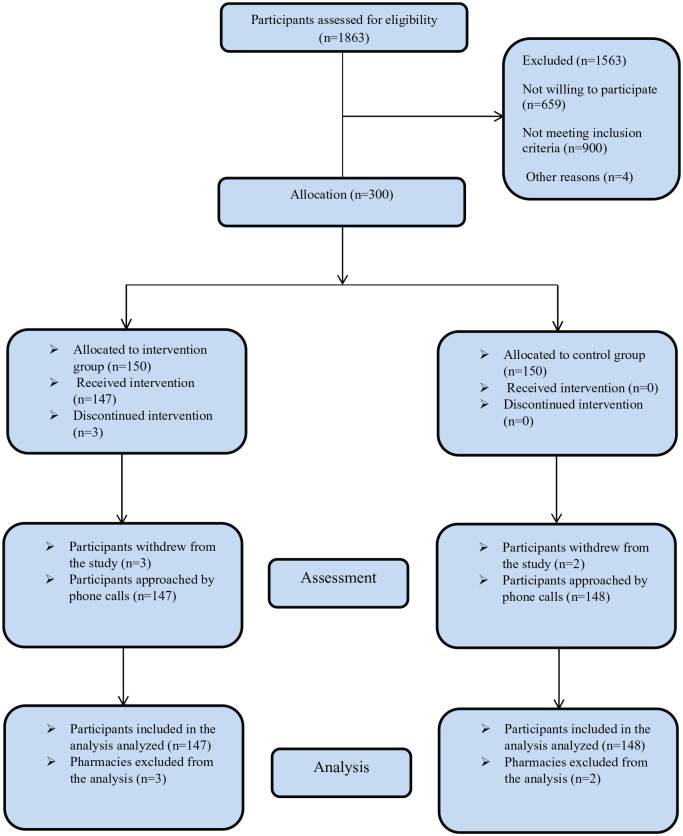
The flow of the study.

Between January 03 and March 25, 2021, a randomized-controlled trial was conducted in Jordan, where participants who matched a set of criteria were randomly assigned into two groups (1:1 ratio). The active group received 12 pharmacist-based virtual coaching sessions; a session per week. Participants allocated to the control group received no coaching. At the end of the last coaching session, both groups were asked to complete a structured questionnaire for outcome assessment. Participants in the active group were followed up to 2 weeks after the end of the last coaching session. The trial protocol and procedures were adopted from the Consolidated Standards of Reporting Trials (CONSORT 2010) (Figure 1). The research ethics approval was given by the Institutional Review Board at the University of Petra.

To calculate the sample size, we reviewed the findings of controlled trials aimed to test pharmacist-based health coaching.^7,20^ It was assumed that the intervention will lead to approximately 20% change in participants’ behaviors. Taking the level of significance as 5%, the power as 80%, and the drop-out rate as 10%, the sample size was 150 participants from each group based on a formula adopted from Rosen’s paper.^21^

### Participants

To recruit 20 pharmacists, we needed to approach 67 pharmacists from 49 community pharmacies in four different Jordanian regions using proportionate random sampling. It was important to recruit pharmacists with substantial experience (more than 6 years) and those who were working in pharmacies that were well-known and easily accessible to the public. Workload, lack of interest, inadequate staffing, concern of contracting the virus, and absence of financial incentives were all barriers toward engagement of pharmacists in this trial. In these cases, we approached the nearest eligible pharmacist.

Each pharmacist included in the study was asked to recruit 15 participants. Recruitment of participants was conducted over 2 phases. First, for eligibility screening, a structured questionnaire was randomly delivered to the public (n = 1863) by the recruited pharmacists. Second, based on public’s responses and with the purpose of recruiting 300 participants, pharmacists needed to approach 963 individuals and ask them to participate in the study. Those participants were adults older than 18 years of age, disbelieving in COVID-19, unwilling to receive COVID-19 vaccines, and usually not taking any precautionary measures (such as wearing masks, avoiding social gatherings, keeping distance) Participants, who were already infected with COVID-19, have taken COVID-19 vaccine, or were part of an ongoing trial, were excluded from the study. Those, who matched the above criteria and accepted participation in the study, were asked to sign a consent form and were informed that their information would be kept coded and confidential, and also, they had the right to withdraw from the study anytime. Allocation of participants into either active or control groups was carried out using the random number generator in the Excel (Microsoft Corp., Redmond, WA). Pharmacists, participants, and the statistician were blinded to trial arm assignment. Nonetheless, research associates were not.

### Pharmacist Training

Pharmacists included in the study were given intensive training on research method (recruitment and dealing with participants) and health coaching principles and techniques. Based on geographic location, pharmacists were divided into 4 groups, of which each group received 1 face-to-face and 2 virtual training workshops. The training was delivered by the principle investigator (an expert coach, hosted more than 500 times by local, regional, and international TV channels, and by local and regional governmental and private hospitals to train medical staff during the pandemic). The content of the training was rich and interactive, and included discussions about health coaching, communication skills, and the concept of behavioral change process in light of self-determination theory, self-concordance theory, the transtheoretical model, and the COM-B model.^22-24^ The COM-B model emphasizes that for any behavioral change to occur, 3 major factors are needed: (1) Capability, (2) Opportunity, and (3) Motivation. The coach (DAQ) explained the importance of authentic communication between the pharmacist and the participant by instructing pharmacists to practise facilitative listening to the participants’ verbal and non-verbal communication. The 4 Pillars of Health Coaching mentioned in Neuner-Jehle’s paper^25^ were adopted, tailored, and presented to pharmacists during the training workshops. During the training period, the trial protocol, the outlines, and the instructions of health coaching sessions were delivered to pharmacists.

### Pharmacist Virtual Health Coaching to Participants

Due to the nature of the pandemic and the seriousness of the situation, it was decided that health coaching sessions to participants should be conducted intensively 3 times per week over a month. Participants assigned to the active group were invited into pre-scheduled online meeting sessions. To improve pharmacist-participant relationship, each pharmacist was assigned to coach the participants he or she initially approached and recruited. Pharmacists were instructed to provide all sessions via virtual coaching using Zoom^®^ software (Zoom Inc, California, USA) for 20 minutes per each session. Many topics were presented for discussion. First, pathways of COVID-19 transmission, which include inhaling respiratory droplets containing the infection or touching eyes or nose with infected hands. Second, ways to block the transmission pathways, which include social distancing, immunization, frequent hand washing, cleaning surfaces, and other measures. Healthy habits and precautionary measures that could stop the spread of the virus were discussed almost in every session. Third, pathogenesis and severity of COVID-19. Fourth. long-term effects of COVID-19 or “long COVID-19,” which includes loss of smell and taste, headache, general fatigue, heart palpitations, and others. Additionally, the types and benefits of vaccines available were also discussed. The coaching sessions focused on each individual’s role in the pandemic control, and how each participant can be an influencer to put an end to the pandemic. Pharmacists provided emotional support for those participants who lost their loved ones or jobs due to the pandemic.

### Outcome Assessment and Measures

A data reporting form was developed after taking a deep look into the literature. Pharmacists approached participants from both arms using phone calls and filled the form at two points; (1) at baseline (before the first session), and (2) at the end of the last session. The data reporting form included questions about the daily behaviors that could block the pathways of COVID-19 transmission. More specifically, participants were asked if they frequently washed their hands, used disposable tissue, cleaned surfaces, avoided touching and shaking hands, avoided social gathering (such as weddings and funerals), avoided unnecessary travels, and isolated themselves when infected. Also, participants’ willingness to receive the COVID-19 vaccine was assessed. Two weeks after completing the coaching sessions, pharmacists approached participants again using phone calls and asked if they or one of their family members contracted COVID-19.

### Data Management and Analysis

Each pharmacist was asked to enter the data collected from the phone calls into an Excel sheet (Microsoft Corp., Redmond, WA), all sheets were compiled into a single dataset, and then data in the dataset were compared with original forms by the investigators. The final version of the dataset was entered into the SPSS software version 26.0 (IBM Corp., Chicago, IL) for statistical analysis. Categorical variables were presented as absolute values with proportions, and continuous variables were presented as mean values with standard deviation. For the comparison of variables between the active and the control groups, the χ^2^ test, or Fisher’s exact test, and Mann-Whitney *U* test were used as appropriate the significance level was defined as *P* < .05. Univariate logistic regression was used to find significant determinants of not getting COVID-19 infection after receiving pharmacist-based health coaching. The dependent variable for the regression model was infection status (infected vs not infected), while independent variables were all behaviors assessed at the end of coaching sessions. The reference for independent variables (behaviors) was using face masks appropriately. It was selected based on participants’ responses to a question in screening questionnaire about the most common COVID-19-induced behavior that influenced their daily life (face masks).

## Results

Of the 300 participants who gave their consent for participation, 295 completed the study (147 from the active arm and 148 from the control arm). Three participants withdrew from the active group and 2 from the control group. Overall, more than half (54.91%) of the participants were females and around one-third (30.84%) were married. Our descriptive analysis showed that although more than half (55.94%) of the participants had a college degree, more than one-third (34.23%) were unemployed. Socio-demographic information did not significantly differ between the 2 arms (Table 1).

**Table 1.** Sociodemographic Characteristics of Participants Included in the Analysis.^a^

Characteristics	Total (n = 295)	Health coaching arm (n = 147)	Control arm (n = 148)
Age, years, mean (SD)	34.97 (10.06)	35.87 (9.17)	34.08 (10.96)
Gender, female	162 (54.91)	78 (53.06)	84 (56.75)
Marital status, married	91 (30.84)	47 (31.97)	44 (29.72)
Born outside Jordan	8 (2.71)	5 (3.40)	3 (2.02)
Employment status			
Employed full time	109 (36.94)	53 (36.05)	56 (37.83)
Employed part time	67 (22.71)	32 (21.76)	35 (23.64)
Unemployed	101 (34.23)	53 (36.05)	48 (32.43)
Retired/disabled/other	18 (6.10)	8 (5.44)	10 (6.75)
Education			
Below high school	54 (18.30)	31 (21.08)	23 (15.54)
High school	76 (25.76)	36 (24.48)	40 (27.02)
College degree	165 (55.94)	81 (55.10)	84 (56.76)
Income (annually)			
<8000$	176 (59.66)	86 (58.2)	90 (60.81)
8000-15 000$	95 (32.20)	47 (31.97)	48 (32.43)
>15 000$	24 (8.13)	13 (8.84)	11 (7.43)
Regular smoker, yes	111 (37.62)	55 (37.41)	56 (37.83)
Chronic condition, yes	66 (22.37)	31 (21.08)	35 (23.64)

*Note.* Data are presented as n (%) unless otherwise stated. SD = standard deviation; $ = American dollar.

a No significant differences between the study’s arms.

The proportions of using face masks, avoiding crowds, and the willingness to be isolated when infected in the active arm were significantly increased from 51.70%, 53.74%, and 59.86% at baseline to 91.83%, 80.27%, and 96.59% at the end of coaching sessions, respectively (*P*-value less than .05 for all) (Table 2). In addition, the proportions of behaviors, such as disinfecting surfaces, not touching T-zone, and avoiding sharing personal belongings with colleagues at work were increased from 36.05%, 27.89%, and 46.93% at baseline to 63.94%, 52.38%, and 87.75% at the end of coaching sessions, respectively (*P*-value less than .05 for all). Nonetheless, the increase in the proportion of avoiding unnecessary travels in the active arm was not significant (15.64% at baseline vs 26.53% at the end of coaching sessions, *P* = .69). On the other hand, all differences between the proportions of behaviors at baseline and at the end of the study in the control arm were insignificant.

**Table 2.** Findings Related to Changes in Behaviors Over the Study Course.

Parameters	Health coaching arm (n = 147)			Control arm (n = 148)		
	At baseline	At the end of the fourth session	*P* value	At baseline	At the end of the fourth session	*P* value
Using face masks as appropriate	76 (51.70)	135 (91.83)	**.001**	68 (45.94)	72 (48.64)	.18
Frequent hands washing with soap or sanitizer	112 (76.19)	129 (87.75)	**.008**	115 (77.70)	123 (83.10)	.26
Disinfecting surfaces and objects	53 (36.05)	94 (63.94)	**.003**	51 (34.45)	54 (36.48)	.34
Avoid touching T-zone	41 (27.89)	77 (52.38)	**.013**	44 (29.72)	45 (30.40)	.042
Maintaining physical distance	64 (43.53)	116 (78.91)	**.009**	67 (45.27)	67 (45.27)	1.00
Using disposable tissue	108 (73.46)	139 (94.55)	**.002**	112 (75.67)	111 (75.00)	.76
Avoid unnecessary travel between cities	23 (15.64)	39 (26.53)	.069	26 (17.56)	27 (18.24)	.61
Avoid crowds	79 (53.74)	118 (80.27)	**.027**	75 (50.67)	77 (52.02)	.13
Avoid sharing personal belongings with family members	33 (22.44)	107 (72.78)	**.003**	28 (18.91)	34 (22.97)	.30
Avoid sharing personal belongings with colleagues at work	69 (46.93)	129 (87.75)	**.004**	60 (40.54)	64 (43.24)	.09
Willing to isolate myself if advice to	88 (59.86)	142 (96.59)	**.02**	85 (57.43)	93 (62.83)	.07

*Note.* Data are presented as n (%) unless otherwise stated. *P* value from χ^2^ test, bolds indicate significant *P* values.

There were three significant predictors for not getting the infection (Table 3); not touching the T-zone (OR = 0.43; 95% CI, 0.24-0.89), using disposable tissues (OR = 0.30; 95% CI, 0.18-0.77), and avoiding sharing personal belongings with colleagues at work (OR = 2.22; 95% CI, 1.74-3.09).

**Table 3.** Association of Behaviors of Health Coaching Arm Reported at the End of the Fourth Session with Infection Status (Got vs Not Got Infected).^a^

Parameters reported at the end of the fourth session	Health coaching arm (n = 147)		
	Infected/close family member infected	Not infected/not a close family member infected	Predicting “not got infected/not a close family member got infected,” OR (95% CI)
Use face masks as appropriate (ref)	11 (8.14)	124 (91.85)	1.00
Frequent hands washing with soap or sanitizer	17 (13.17)	112 (88.18)	0.58 (0.34-1.66)
Disinfect surfaces and objects	8 (8.51)	86 (91.48)	0.95 (0.49-4.94)
Do not touch T-zone	13 (16.88)	64 (83.11)	**0.43 (0.24-0.89)**
Maintain physical distance	2 (1.72)	114 (98.27)	5.05 (0.80-8.99)
Using disposable tissue	31 (22.30)	108 (77.69)	**0.30 (0.18-0.77)**
Avoid unnecessary travel between cities	13 (32.55)	26 (67.44)	0.16 (0.10-2.63)
Avoid crowds	13 (11.01)	105 (88.98)	0.74 (0.55-2.41)
Avoid sharing personal belongings with family members	14 (13.08)	93 (86.91)	0.59 (0.38-1.62)
Avoid sharing personal belongings with colleagues at work	5 (3.87)	124 (96.12)	**2.22 (1.74-3.09)**
Willing to isolate myself if advice to	12 (8.45)	130 (91.54)	0.95 (0.46-2.88)

*Note.* OR = odds ratio; Ref = references; CI = confidence interval; Bold values indicate significant results.

a Univariate logistic regression, where probability of each behavior was modeled for infection status (got infected vs not got infected).

**Figure 2.**
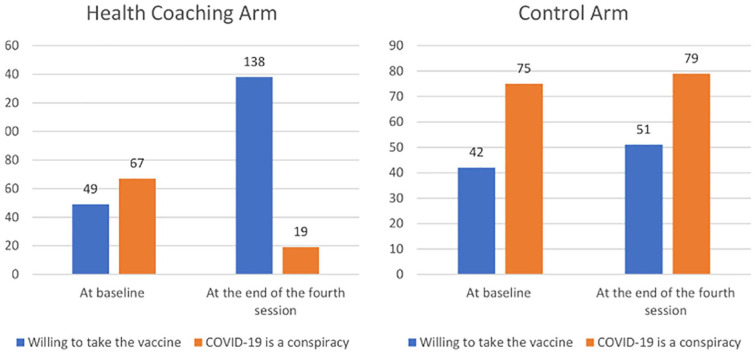
Participants’ perception about COVID-19 and willing to take the vaccine by study arms.

The proportion of participants in the active and control arms who thought COVID-19 was a conspiracy was changed from 45.57% and 50.67% to 12.90% and 53.37%, respectively. The proportion of participants in the active arm who were willing to receive COVID-19 vaccines was increased from 33.33% at baseline to 93.87% at the end of coaching sessions (*P* = .001); however, it was not significantly increased in the control arm (28.37% at baseline vs 34.45% at the end of coaching, *P* = .089) (Figure 2).

## Discussion

The current situation in many places around the world, especially in low and middle-income countries, is a fast spreading pandemic faced by overwhelmed healthcare services, poor public engagement in preventive measures, and a low number of vaccine doses. The findings of this study indicated that pharmacist-based health coaching, even via virtual coaching, could improve people’s behavior blocking pathways of COVID-19 transmission and increasing their acceptance for vaccination. To our knowledge, this study was the first of its kind to assess the impact of pharmacist-based virtual health coaching on behaviors related to COVID-19. This study may offer a potential strategy that could curtail the spread of the virus by encouraging and educating people how to practise daily life activities safely in times of COVID-19.

The strategy implemented in this study led to more adherence to public health measures such as using face masks in public places, avoiding crowds, avoiding touching T-zone, avoiding sharing personal belongings with colleagues at work, and others. Our findings could be attributed to several factors. First, we believe that not only was directing participants toward the importance of certain behaviors in curtailing the spread of the virus the reason behind convincing more participants to use face masks for instance, but also more importantly coaching them on how real and serious the virus is. Second, pharmacists were taught during the training session that knowledge-based coaching alone would not enhance behavioral change,^26^ and knowledge should be adapted to each participant’s life style. This was achieved by using scenarios built based on daily life experiences. How to act if someone invited you to a wedding without precautionary measures? How to act if your colleague at work wanted to borrow your headphones? How to act if your cousin got infected and you had been in close contact with him the day before? How to behave if you got infected while you were living with your grandmother? Upon discussing these scenarios, pharmacists were instructed to consider mental, physical, and emotional aspects of the scenarios. Third, pharmacists were instructed toward finding motivations that could drive people to change. In our case, economic recovery and safety of the family were considered by pharmacists as core motivations that drove an individual to change.

Surprisingly, avoiding unnecessary travel between cities was not significantly increased after coaching sessions. The plausible explanation to this finding was that such a behavior change cannot be sustained, because cities in Jordan are geographically close to each other and many people have different cities for living and working. Therefore, without implementation of movement restrictions, changing this behavior seemed improbable. Our finding was in line with the recommendations delivered by Lonie et al^7^ which suggested that in order to achieve an effective health coaching, behavioral change to be suggested in the process should be sustainable and adaptable in an individual’s daily life.

Our findings suggested that using face masks had a great potential to prevent the spread of COVID-19. Face masks may provide dual protection for those who use them and others around them. A recent review showed that using face masks offered a double barrier against the spread of the COVID-19.^27^ Our findings were also in line with a German study which concluded that face covering can assist in reducing the growth of COVID-19.^28^

Interestingly, avoiding sharing personal belongings with colleagues at work considerably reduced the probability of getting the infection. Jordan as many developing countries lacks active remote public services, and paper transactions are still widespread. This highlighted the urgent need to implement contactless interaction and electronic tools in public services, not only to limit face-to-face interaction between employees and clients, but also between employees themselves, because, in many workplaces in Jordan, employees used to share the same printers, prayer carpets, meals, and cooking utensils. However, although individual-based efforts are important to actively maintain contactless interaction among colleagues at work, a system approach that provides both policies and tools facilitating such behavior is crucial.

Because recent studies showed a high prevalence of COVID-19 vaccine hesitancy associated with conspiracy beliefs among Jordanians,^29,30^ our research focused on finding a trusted source of information that could correct many misconceptions, ease participants’ fears and urge them to receive the vaccines. Accordingly, our findings indicated that pharmacist-based health coaching increased the proportion of participants in health coaching arm who were willing to take COVID-19 vaccine.

### Limitations

This study had several limitations that should be addressed. First, our findings were subjected to participants’ bias, as we used self-reporting for outcome assessment, which could lead to a reduced reliability of the data. Many participants might have seen it difficult or embarrassing to report accurately on their daily behavior. Nonetheless, it was not feasible to conduct an observational study given the COVID-19 situation. Thus, we tried to minimize the impact of this limitation by ensuring confidentially and remove communication barriers between pharmacists and participants. Second, given that participants’ behaviors were assessed just at two points (at baseline and after the end of coaching sessions), better understanding of behavioral change of participants would have been reached if participants’ behaviors were assessed after each session. However, this method was the initially planned, and due to practical reasons, it was modified to the current version of outcome assessment. Third, there may have been some differences in baseline information between the two arms, which could have affected the findings of the between-arms comparisons. Fourth, participants belonging to the control arm were not followed to track if they got the virus or not.

## Conclusion

Pharmacist-based virtual health coaching could be a potential strategy to increase the proportion of behaviors that could curtail the spread of COVID-19. For example, this strategy increased participants’ acceptance of immunization. Using face masks and avoiding sharing personal belongings with colleagues at work were considerably associated with preventing COVID-19 infection.
